# The Angle of Progression at Station 0 and in Magnetic Resonance and Transperineal Ultrasound Assessment

**DOI:** 10.1155/2015/748327

**Published:** 2015-09-21

**Authors:** D. Iliescu, S. Tudorache, R. Dragusin, O. Carbunaru, C. Patru, M. Florea, I. A. Gheonea

**Affiliations:** ^1^Department of Obstetrics and Gynecology, Prenatal Diagnostic Unit, University of Medicine and Pharmacy Craiova, Petru Rares 2, 200349 Craiova, Romania; ^2^Department of Obstetrics and Gynecology, University Emergency County Hospital Craiova, Romania, Tabaci 1, Dolj, 200642 Craiova, Romania; ^3^Department of Radiology, University of Medicine and Pharmacy Craiova, Petru Rares 2, 200349 Craiova, Romania

## Abstract

The transperineal ultrasound (TPU) value of the angle of progression (AOP) during fetal head engagement, at station 0, is a critical cut-off for current obstetrical practice, especially when intrapartum instrumental interventions are required. Still, controversial measurements were reported in previous high resolution imagistic studies. Our TPU and direct “gold-standard” magnetic resonance (MRI) measurements confirm that station 0 corresponds to a 120° AOP, concordantly. Based on these findings, the fact that an AOP of 120° or greater was previously strongly associated with vaginal delivery may be due to the achievement of head engagement in labor.

## 1. Introduction

The angle of progression (AOP) measured by transperineal ultrasound (TPU) was demonstrated as an objective, accurate, and reproducible indicator of the fetal head descent during labor, superior to clinical obstetrical evaluation, and an AOP of 120° or greater was associated with subsequent vaginal delivery [1, 2]. The advantages of this precise evaluation technique triggered high resolution computed tomography (CT) [3] and magnetic resonance imaging (MRI) [4, 5] studies that aimed to indicate the AOP corresponding to fetal head engagement (0 station), but the results were discordant: 99° and 120°, respectively. Moreover, there are some important limitations of these studies. CT [3] data were obtained from nonpregnant women and by using bony landmarks, unlike the TPU measurements that use the symphyseal nonbony capsular tissue as a landmark. Also, the correlations between CT and MRI measurements with TPU evaluations were based on statistical assumptions only, as none of the fetal heads was engaged in the studied groups [4, 5]. The authors admitted the limitations and highlighted the necessity of future research to confirm the data by measuring directly the AOP during engagement of the fetal head in the maternal pelvis.

The case presented is part of an ongoing research meant to study the correlation of the measurements performed by transperineal ultrasound and the gold-standard MRI technique. Also, we correlate the measurements with the fetal head station measured by MRI.

Ethics approval of the study protocol was obtained from the Ethics Committees of the University. The operator is responsible for explaining the procedures and obtaining an informed written consent from all women accepting to take part in the study.

## 2. Case Report

**Figure 1:**
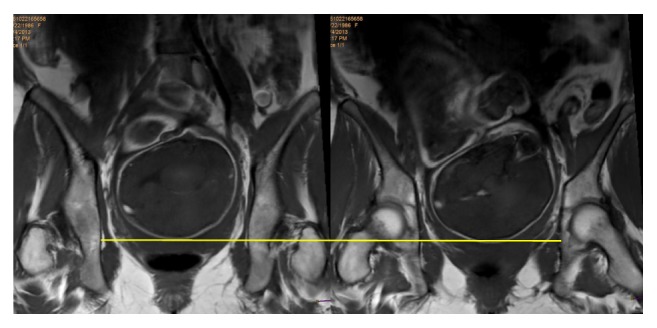
MRI coronal planes showing the fetal head at the level of the ischial spine.

**Figure 2:**
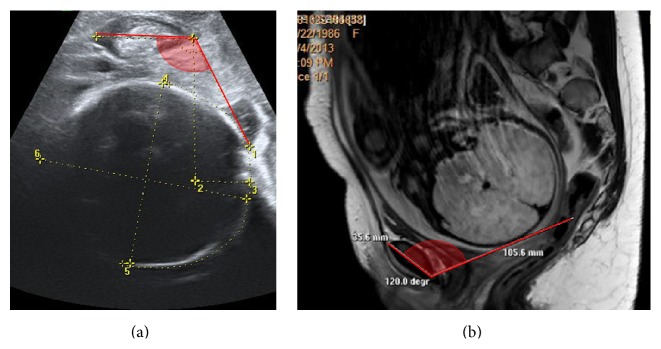
Measurement of the AOP in sagital TPU (a) and MRI (b) views, showing similar values, 120°.

A 26-year-old term (39GW) primipara with singleton eutrophic fetus (3440g) was clinically found with engaged fetal head at the routine pregnancy care clinical examination. We performed MRI on a high-field scanner with high resolution parallel pelvic images and short time acquisition sequences (Ingenia 3.0T, Philips Healthcare) using a body coil. The woman was examined in the supine position, with legs flexed and empty bladder. The imaging algorithm included T1- and T2-weighted TSE (turbospin echo) sequences in coronal, sagittal, and axial planes with the following settings: time of echo (TE): 8 ms, time of repetition (TR): 529 ms, and thickness: 4. Offline, we investigated the correlation between the leading part of the fetal skull and the interspinal plane that confirmed station 0 (Figure 1), and we measured the AOP in sagittal plane (120°, Figure 2).

TPU was performed immediately after the MRI evaluation in the same posture, in order to avoid fetal head movements between the evaluations. A Voluson 730 Expert system equipped with a 4–7 MHz transabdominal 3D transducer (GE Healthcare Ultrasound) was used. Occipitoanterior position was determined transabdominally [6] and several measurements of the AOP [1] were recorded for offline measurement, which confirmed similar results, 118–121° (Figure 2).

## 3. Discussion and Conclusion

The importance of our research is the direct quantification of the AOP using both TPU and the gold-standard MRI technique at station 0. This AOP cut-off, still controversial in previous imagistic studies, is critical for current obstetrical practice, and especially when intrapartum instrumental interventions are required, because it offers a rapid and precise determination of the fetal head engagement.

In this case, MRI results confirmed that station 0 corresponds to an AOP of 120°, as indirectly estimated before in a study [4], based on statistical calculations. The TPU and MRI measurements were concordant with the engagement of the fetal head, as previously reported for higher pelvic stations [4].

In our opinion, the fact that an AOP of 120° or greater was strongly associated with vaginal delivery [1, 2] may be due to the achievement of head engagement in labor.

Although our findings are very important for labor ward practice, they are based on the evaluation of only one patient. Therefore, we consider that their implementation should be preceded by confirmation in larger groups.
